# Detection of helminths by loop-mediated isothermal amplification assay: a review of updated technology and future outlook

**DOI:** 10.1186/s40249-019-0530-z

**Published:** 2019-03-25

**Authors:** Miao-Han Deng, Lan-Yi Zhong, Okanurak Kamolnetr, Yanin Limpanont, Zhi-Yue Lv

**Affiliations:** 1 Zhongshan School of Medicine, Sun Yat-sen University, Guangzhou, 510080 China; 2 Key Laboratory of Tropical Disease Control (Sun Yat-sen University), Ministry of Education, Guangzhou, 510080 China; 3 Faculty of Tropical Medicine, Mahidol University, Bangkok, 10400 Thailand; 4 Provincial Engineering Technology Research Center for Biological Vector Control, Guangzhou, 510080 China; 5 Fifth Affiliated Hospital, Sun Yat-sen University, Guangzhou, 519000 China

**Keywords:** Loop-mediated isothermal amplification, Helminth, Point-of-care-test, Epidemiological surveillance, Field survey

## Abstract

**Background:**

Helminths are endemic in more than half of the world’s countries, raising serious public health concerns. Accurate diagnosis of helminth infection is crucial to control strategies. Traditional parasitological methods, serological tests and PCR-based assays are the major means of the diagnosis of helminth infection, but they are time-consuming and/or expensive, and sometimes provide inaccurate results. Loop mediated isothermal amplification (LAMP) assay, a sensitive, simple and rapid method was therefore developed for detection of helminths. This study aims to discuss the current status of application of LAMP on helminths detection and to make a comprehensive evaluation about this updated technology and its future outlook by comparing with several other diagnostic methods.

**Main body:**

This review summarizes LAMP assay applied for helminth detection and helminthiasis surveillance. The basic principle of LAMP is introduced to help better understand its characteristics and each reported assay is assessed mainly based on its detection sensitivity, specificity and limitations, in comparison with other common diagnostic tests. Moreover, we discuss the limitations of the assays so as to clarify some potential ways of improvement.

**Conclusions:**

Here, we summarize and discuss the advantages, disadvantages and promising future of LAMP in heliminth detection, which is expected to help update current knowledge and future perspectives of LAMP in highly sensitive and specific diagnosis and surveillance of helminthiasis and other parasitic diseases, and can contribute to the elimination of the diseases from endemic areas.

**Electronic supplementary material:**

The online version of this article (10.1186/s40249-019-0530-z) contains supplementary material, which is available to authorized users.

## Multilingual abstracts

Please see Additional file 1 for translations of the abstract into the five official working languages of the United Nations.

## Background

Helminths, including trematodes (flukes), nematodes (roundworms) and cestodes (tapeworms), are associated with substantial morbidity and economic losses worldwide [1–3]. Approximately one-sixth of the worlds’ population is infected with helminths [4], with an estimated 15 billion individuals, particularly in low socio-economic regions, suffered from soil-transmitted helminth (STH) infections [5, 6]. Although most of helminths have been well investigated epidemiologically [7], actual distributions of them are still unknown and accurate diagnosis is urgently needed because of their generally non-specific and similar symptoms (nausea and/or vomiting, diarrhoea, abdominal pain, and fever) between the causative species [8, 9].

The approaches to clinical diagnosis and epidemiological surveillance of helminthiasis vary according to the samples, infectious stages, life cycle, morphological characteristics of helminths. Although the methods are diversified, there is not an ideal and reliable point-of-care (POC) diagnostic method that can eminently meet the World Health Orgnization (WHO)’s expectation of characteristics of affordable, sensitive, specific, user-friendly, rapid and equipment-delivered (ASSURED) [10, 11]. Though the simple and cost-effective morphological identification of parasites has been commonly employed in clinical diagnosis and field survey, it shows poor sensitivity in low-density parasite infections [12–16]. Furthermore, with respect to the discernment of parasite eggs that are morphologically similar, it will lose its specificity [12–16]. In addition, its prerequisite for a considerable quality and quantity of manpower also makes it unadaptable as a POC tool [17]. To avoid misdiagnosis and missed diagnosis, particularly in low-grade infections and in low-intensity regions, enzyme-linked immunosorbent assay (ELISA), as a representative of serological tests, has been applied [18, 19]. However, the major drawbacks with the use of ELIAS are clear due to its inability to distinguish between past and present infections, relatively high false-positive rate, and cross reactions [16, 19, 20]. Alternatively, a series of polymerase chain reaction (PCR)-based techniques, which are both specific and sensitive, started a new era for nucleic acid-based molecular detection of helminths. The 1990s witnessed the inception of various amplification techniques, e.g., nucleic acid sequence-based amplification [21], strand displacement amplification [22], and rolling circle amplification [23]. But none of these methods manages to conquer the inherent weakness of heavy dependence on a particular instrument or elaborate detection methods [24, 25]. As a result, their application is restricted where they are urgently needed, such as in primary medical institutions, underdeveloped areas and field studies [16, 26, 27]. As LAMP, a nucleic acid amplification method with extremely high sensitivity and specificity, appears to promise an appealing resolution for almost all of issues mentioned above, this review investigates the recent research progress in use of LAMP in helminth detection and make an comprehensive evaluation about this updated technology and highlights the future perspectives regarding the possible applications of LAMP in diagnosis of parasitic diseases, comparing with etiological detection, serological tests and other molecular assay.

In the present paper, we reviewed published studies between 2001 and 2018 to identify studies exploiting LAMP in helminth detection. A comprehensive search strategy was developed in PubMed, proper key words and free text terms employed. Search terms were “(helminth” [All fields] OR nematode [All fields] OR cestode [All fields] OR trematode [All fields]) AND (“loop-mediated isothermal amplification” [All fields] OR “LAMP” [All fields]). In brief, information was collected and analyzed from 54 articles in Chinese or English.

## Main text

### Principle of LAMP

**Fig. 1:**
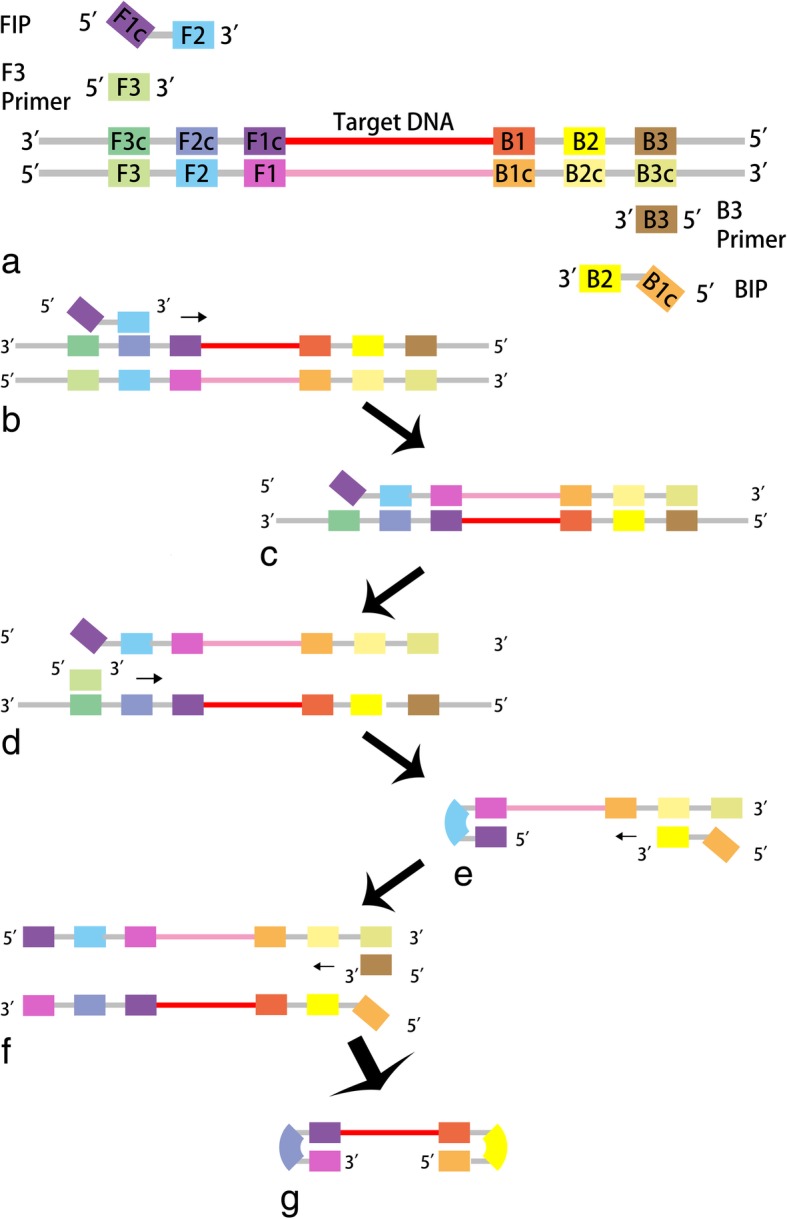
Principle of LAMP. The initiative stage of LAMP assay: Besides the target DNA, the reaction system in (**a**) contains a set of inner primers—BIP and FIP, and a set of outer primers –F3 and B3 primer. An inner primer initiates the reaction in (**b**-**g**) by replacing the template strand with the help of polymerase with strand-displacement activity such as Bst DNA polymerase. An outer primer working, a single strand DNA is released, serving as the template of the following reaction. The similar strand-displacing DNA synthesis proceeding in the other end, yields the dumbbell-like DNA strands with a stem-loop structure in (**g**), which take part in the auto-cycling stage

**Fig. 2:**
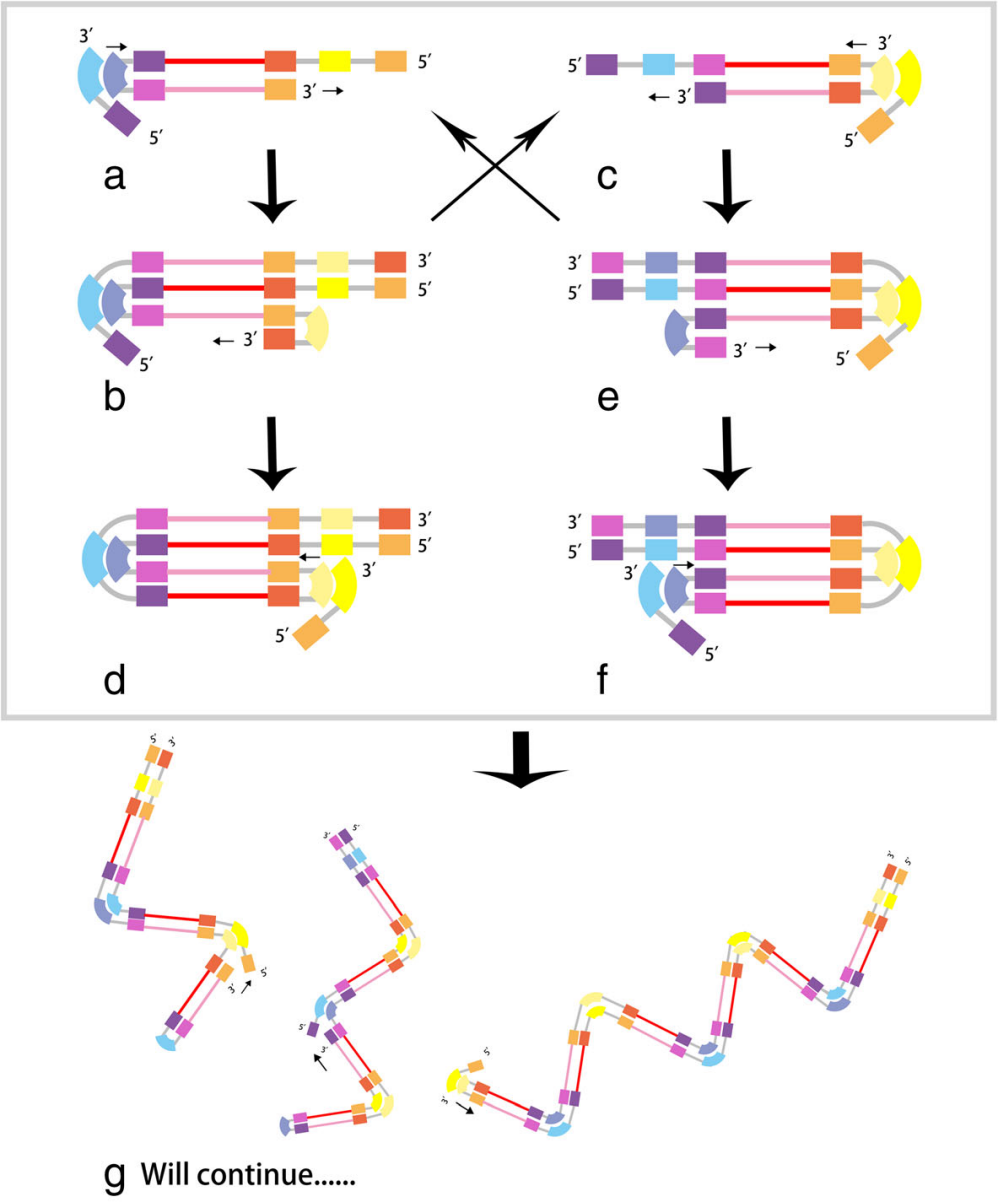
Principles of LAMP assay. The auto-cycling stage: After the self-hybridizing reaction dissociate the stem-loop structure in the 5′ end, an inner primer hybridized to the stem-loop in the 3′ end, initiating the auto-cycling stage. The newly synthesized 3′ end continues its self-hybridizing reaction, producing a stem-loop DNA essentially identical with the initial one and a new one with the stem twice as the original one. Inner primers hybridizes, elongating new strands once there is a stem free thus to repeat the aforementioned reaction. The final products in (**g**), namely stem-loop DNAs of varied sizes and cauliflower-like structures with multiple loops, accumulates as long as the reaction circularly continues

Using the sophisticated mechanism of auto-cycling strand displacement DNA synthesis, LAMP was developed as a novel method requiring minimal instrumentation [25]. An inner primer, termed forward inner primer (FIP), containing sequences corresponding to the sense and antisense sequences of the target DNA, initiates the reaction [25]. An outer primer primes the subsequent strand displacement DNA synthesis [25]. As a result, a single-stranded DNA molecule is released, serving as the template for similar DNA synthesis primed by another set of primers at the other end of the target DNA [25]. In the initial step, dumbbell-like DNA strands with a stem-loop structure are produced (Fig. 1) [25]. In the following cycling step, DNA synthesis is triggered by an inner primer hybridizing to the loop on the product, which produces an identical stem-loop structure [25]. Released by the strand displacement reaction, the 3′ end of the original stem-loop DNA molecule is able to complete the self-primed DNA synthesis, yielding a new stem-loop DNA molecule with the stem twice length as the original one [25]. The above reactions circularly repeat during the entire cycling step (Fig. 2) [25].

Without a thermocycler [28], target DNA is amplified by employing *Bst* DNA polymerase under a constant temperature of 60–65 °C, and accumulated 10^9^ copies of target DNA in less than an hour, with a detection limit of a few copies [24, 25, 29]. Properly designed primers are given [30], as four different primers recognize 6 distinct sequences in a target DNA. The process will be blocked once non-specific recognition occurs, hence high selectiveness [29]. If supplemented with loop primers, stem primers and swarm primers, an even higher reaction speed can be expected [31–33]. The final products of the LAMP reaction are stem-loop DNAs inverted with a large amount of repeats of the target and cauliflower-like structures with multiple loops. The approaches of endpoint monitoring differ according to varied purposes. Sometimes agarose gel electrophoresis is employed as the gold standard, but it is not always compulsory [25, 34, 35]. And turbidity determination is more suitable for field research [24]. As a pyrophosphate ion is released once a nucleotide is added to the DNA strands, a large number of target DNA will be accumulate by the end of the assay, forming visible white precipitates of magnesium pyrophosphate, which is used to determine whether the target nucleic acid was amplified or not [36]. Based on the principle mentioned above, LAMP is characteristically able to meet the ASSURED needs, since it is a one-step process running within 1 h when there is *Bst* polymerase and a simple heating block, and the result can be read by the naked eyes. Furthermore, LAMP has also reported to be more tolerant than PCR for some biological inhibitors. Therefore, it can detect DNA in some specific clinical samples, such as swabs, without DNA extraction [28].

For further improvement, the fluorescent probe calcein, DNA-binding dye SYBR Green I, DNA-functionalized gold nanoparticles, etc., are then added to reach a higher sensitivity [37, 38]. To achieve the analysis of minute quantities of nucleic acid, real-time turbidimetry [39] is used, followed by the introduction of cationic polymers, e.g., poly-ethylenimine, which makes it feasible for use on a conventional UV illuminator [40]. Further progress is based on colorimetry with a hydroxy naphthol blue (HNB) indicator, which changes colour without affecting the amplification reactions and can be performed in a microtiter plate [35, 41], which empowers its development as a portable tool in field surveys. Combined with several biotechnology tools, LAMP has been widely applied in recent years, e.g., LAMP-chromatographic lateral flow dipsticks [42] and LAMP-ELISA [43, 44]. Moreover, simultaneous amplification of multiple targets has been achieved, termed multiplex LAMP (mLAMP), and has currently attracted much attention in the biomedical applications [45, 46].

Nevertheless, as disadvantages are always accompanied by advantages, LAMP assays still have a long way to go until their robustness, performance and utility are validated [47]. As mentioned above, primer design is a prerequisite and critical part of the LAMP assay [48], but it is also a major drawback plaguing researchers, even helpful software can be freely acquired [49–51]. The introduction of multiple primers theoretically promotes specificity, whereas it may increase the risk of primer-primer hybridizations, giving rise to template-free amplification at the same time [52]. The chances of false-positive outcomes, however, need further evaluation [45]. To avoid the foregoing situation, redesign of the primers should be taken into consideration [45]. Another chief obstacle is the unintended carryover contamination caused by its extremely high efficacy [48, 53, 54]. An isolated room and a closed reaction system for the test, e.g., agar dye capsule [55], or the pre-addition of dye, hydroxynaphthol blue dye (HNB), are recommended [45]. Another prominent resolution is the emerging lab-on-chip technique, which allows all the analytical steps to be processed on a single chip [56, 57]. Due to the lack of a thermocycler and convenience in sample extraction and endpoint determination, LAMP may prompt the development of lab-on-chip techniques [58, 59]. In combination with LAMP, mLAMP will manifest notable superiority of high-throughput screening, high sensitivity and lower risk of cross-contamination, which shows momentum in multiple target screening and determination of pathogens with frequent gene mutation [46].

### Detection of helminths by LAMP

The impressive progress currently made in the LAMP assay for helminths includes trematodes of *Clonorchis sinensis* [12, 26, 60], *Opisthorchis viverrini* [14, 61, 62], *Amphimerus* spp. [63, 64], *Paragonimus westermani* [15], *Fasciola hepatica* [65–67], *F. gigantica* [65], *Schistosoma japonicum* [16, 27, 68–70], *S. mansoni* [13, 71–77], *S. haematobium* [51, 71, 72, 76]; nematodes of *Necator americanus* [78, 79], *Ascaris lumbricoides* [17, 79]*, Trichuris trichiura* [79], *Toxocara canis* [80] and *T. cati* [81], *Strongyloides stercoralis* [52, 82], *Onchocerca volvulus* [83–86], *Wuchereria bancrofti* [86, 87], *Brugia malayi* [86, 88], *B. tomori* [88], *Loa loa* [89–91], *Dirofilaria repens* [92], *Angiostrongylus cantonensis* [93, 94], *Trichinella spiralis* [95, 96], *Bursaphelenchus xylophilus* [97], and *Haemonchus contortus* [98, 99]; cestodes of *T. solium* [44, 100–103]*, T. saginata* [44, 100–103], *T. asiatica* [44, 100–103], *T. hydatigena* [104], *T. multiceps* [104], *T. pisiformis* [104] and *T. crassiceps* [104], *Echinococcus granulosus* [104–106], *E. multilocularis* [104, 107], *E. equinus* [108], *E. canadensis* [108], *E. felidi* [108], *E. ortleppi* [108, 109] and *E. shiquicus* [104]*,* have been covered in this review for further insight into its adoption for clinical diagnosis, field surveys and surveillance of helminths. The sensitivity and specificity of detection of helminths by LAMP are shown in Table 1.

**Table 1:** The overall information of LAMP assays for helminths

Parasites	Developmental Stages	Samples	Hosts	Target gene	Accession number	Sensitivity	Specificity	Reference
						(Detection Limit)		
Trematodes								
*C. sinensis*	Adult	Bile duct	Cat	Cathepsin B3	AY273803	0.01 ng of DNA/rxn	100%	[60]
	Metacercaria	Muscle	Fish			9 metacercarias/gram		
	Sporocyst, redia, cercaria	Tissue	Snail, shrimp, fish	ITS-2	AF217099	10 fg of DNA/rxn	100%	[12]
						(0.0002 *C. sinensis* per snail)		
	Adult	Bile duct	Rat	Cox1	AF181889	100 fg of DNA/rxn	100%	[26]
	Egg	Stool	Human			10 EPGs		
*F. hepatica*	Adult	–	Cattle, goat, rabbit, sheep, horse	IGS	GU903890	10 fg of DNA/rxn	100%	[65]
	Egg	Stool	Sheep	ITS-2	JF708043	1 pg of DNA/rxn	100%	[66]
					GQ231547			
					JF708026			
					JF708036			
					HM746786			
					AM709622			
					JF432071			
					JF432074			
					JF496714			
					KF425321			
					AM850108			
					HM746788			
					JN828956			
	Egg	Stool	Sheep and cattle	ITS-2	DQ683546, JF824668, KJ200622, AB207148	1 pg of DNA/rxn	100%	[67]
*F. gigantica*	Adult, egg and cercaria	–	Cattle, sheep, buffalo and snail	IGS	GU903891	0.01 pg of DNA/rxn	100%	[65]
*O. viverrini*	Adult	Bile duct	Hamster	ITS-1	EU038151	1 pg of DNA/rxn	100%	[61]
	Cercaria	Tissue	Snail			–	100%	
	Metacercaria	Muscle	Fish			–	100%	
	Adult	Bile duct	Hamster			–	Cross reaction with *O. felineus, F. gigantica* and *Haplorchoihoides* sp., no template-free amplification	[14]
	Egg, Adult and metacercaria	Stool	Human	Nad1	EU443831, DQ882172, DQ882174, EU443833, EU443832, DQ882175, GQ401025, GQ401064, GQ401046, GQ401060, GQ401082, GQ401096, EU022343, EU022346, EU022348, EU022350	1 pg to 100 fg of DNA/rxn	100%	[62]
	Metacercaria and metacercarial cyst	Muscle	Fish			–	100%	
	Egg	Stool	Human			–	100%	
	Adult	Bile duct	Hamster	OvMS6	DQ144069	1 pg of DNA/rxn	100%	[14]
*Amphimerus* spp.	Adult	Liver	Cats and dogs	ITS-2	AB678442.1	1 pg of DNA/rxn	100%	[64]
	Egg	Stool	Human			–	–	
*P. westermani*	Metacercaria	Muscle	Freshwater crab and crayfish	ITS-2	AF159604	0.01 fg of DNA/rxn	100%	[15]
						1 metacercaria/gram		
	Egg	Sputum and pleural fluid	Human			–	100%	
*S. japonicum*	Adult, egg, cercaria	Liver homogenate, stool and serum	Rabbit	SjR2	AF412221	0.08 fg of DNA/rxn	100%	[16]
				SjR2	AY027869			
	–	Serum	Rabbit			positive from 1 week p.i. with 500 cercariae	–	
	Adult	–	–			100 fg of DNA/rxn	Cross reaction with *Sch. mansoni*; no template-free amplification	[69]
	–	Serum	Rabbit			positive from 1 week p.i. with 200 cercariae	–	
	–	Serum	Rabbit	SjR2	AF412221	positive from 3 days p.i. with 30 cercariae	–	[70]
	Miracidium	Tissue	Snail	28S rDNA	Z46504	100 fg of DNA/rxn	100%	[68]
	Miracidium	Tissue	Snail			positive from 1 day p.i with 1 miracidium	–	
						1 infected snail in 100 non-infected snails		
	Miracidium	Tissue	Snail			100 fg of DNA/rxn	100%	[27]
*S. mansoni*	Adult	–	Mouse	Sm1–7	M36086	0.1 fg of DNA/rxn	100%	[71]
	Miracidium	Tissue	Snail			positive from 1 day p.i. with 10 miracidia	–	
	Miracidium	Tissue	Snail			positive from 1 day p.i. with 1 miracidium	–	[72]
	–	Plasma/Serum	Mouse			0.5 fg of DNA/rxn	100%	[74]
						positive from 1 week p.i. with 200 cercariae		
	Adult	–	–	Minisatellite DNA region	L27240	1 fg of DNA/rxn	100%	[73]
	–	Stool	Mouse			positive from 1 week p.i. with 200 cercariae.	100%	
	Adult	–	–	IGS	AJ223842	0.1 fg of DNA/rxn	100%	[75]
	Miracidium	Tissue	Snail			positive from 1 day p.i. with 1 miracidium	100%	
	Miracidium	Tissue	Snail	SmITS	L27240.1, AY446082.1, M63265.1, AF130787.1	70 fg of DNA/rxn	100%	[13]
	Miracidium	Tissue	Snail			1 infected snail in 1000 non-infected snails	100%	
*S. haematobium*	Adult	–	–	DraI	–	0.1 fg of DNA/rxn	100%	[71]
	Miracidium	Tissue	Snail			positive from 1 day p.i. with 5 miracidia	100%	
	Miracidium	Tissue	Snail			positive from 1 day p.i. with 1 miracidium	100%	[72]
	Adult	–	–	18S–28S rDNA	AJ223838	100 fg of DNA/rxn	100%	[76]
	Egg	Urine	Human			25 fg of DNA/rxn	100%	
Nematodes								
*A. lumbricoides*	Egg and adult	Stool	Human	ITS-1	AJ000895	10.8 ng of DNA/rxn	100%	[17]
	Adult	Stool	Human	β-tubulin isotype 1 gene	EU814697	1 pg of DNA/rxn	100%	[79]
*N. americanus*	Egg	Stool	Human	ITS-2	KC896820.1-KC 896825.1, Y11734, AF217891, HQ452515, HQ455217, HQ452537-HQ452543, AJ001599	0.4 fg of DNA/rxn	100%	[78]
	Adult, larva and egg	Stool	Human	β-tubulin isotype 1 gene	EF392851	1 pg of DNA/rxn	100%	[79]
*T. trichiura*	Adult	–	–	β-tubulin isotype 1 gene	AF034219	1 pg of DNA/rxn	100%	[79]
*W. bancrofti*	Microfilaria	Blood	Human	WbLDR	AY297458	0.001 microfilariae/rxn	100%	[87]
	–	–	–			0.00002 microfilaria /rxn	100%	[86]
*Brugian* spp.	–	–	–	Hha I	M12691, AAQA01025653, AAQA01026145, AAQA01018878, AAQA01011954, AAQA01021048, AAQA01005386, AAQA01005790, AAQA01007277, AAQA01004714, AAQA01005124,	1.0 pg of DNA/rxn	Tested positive for both *B.malayi* and *B. timori*	[88]
	Microfilaria	Blood	Feline			0.005 microfilariae/rxn	–	
*B. malayi*	–	–	–			1.0 pg of DNA/rxn	100%	[86]
*O. volvulus*	Adult	Skin nodule	Human	OvGST1a	AF265556.1	0.01 ng of DNA/rxn	100%	[83]
			Black fiy			0.01 ng of DNA/200 insects	–	
	–	–	–			0.01 ng of DNA/rxn	100%	[86]
	Microfilaria	Skin snip	Human	O-150	J04659	0.1 pg of DNA/rxn	100%	[84]
				Cox1	NC_001861.1	100 DNA copies/rxn	Cross reaction with *Ohch. ochengi*	[85]
*Loa loa*	Microfilaria	Blood	Human	LLMF72	HM753552.1	0.2 fg of DNA/rxn	93.00%	[89]
				LLMF342	ADBU02000498.1	0.02 pg of DNA/rxn	(qPCR as gold standard)	
	Adult	Eyes and Blood	Human	LL3M9	M34259.1	0.5 ag of DNA/rxn	100%	[90]
	–	–	–	RF4	JPEI01001237.1 JPEI01001554.1 JPEI01001218.1 JPEI01001588.1 JPEI01001706.1	0.126 pg of DNA/rxn	100%	[91]
	–	Blood	Human			1.6 pg of DNA/rxn	Positive in only 1 of 12 NTCs	
*D. repens*	Larva	Blood	Dog	COI gene	AJ271614, AM749230- AM749234, DQ358814, JF461458, KF692102	0.15 fg of DNA/rxn by real time-LAMP, and 10 fg of DNA/rxn by PI-LAMP	100%	[92]
*S. Stercoralis*(*S. ratti or S. venezuelensis* as laboratory model)	Infective third stage larva	Stool	Human	28S rRNA gene	DQ14570.1	< 10 copies of DNA/rxn < 0.01 of a larva/rxn	100%	[52]
	Infective third stage larva	Stool	Wistar rats	18S rRNA gene	AJ417026.1	Positive from 6 days p.i. with 40 iL3, from 5 days p.i. with 400 iL3 or 4000 iL3	100%	[82]
		urine				Positive from 6 days p.i. with 40 iL3, from 3 days p.i. with 400 iL3 or 4000 iL3	100%	
*A. cantonensis*	The first stage larva	Lung	*snail*	18S rRNA gene	AY295804.1	1 fg of DNA/rxn	100%	[93]
	The third stage larva	Tissue	snail	ITS-1	GU587760.1	0.32 larvae/0.1 g of snail tissue	–	[94]
	Adult	Tissue	snail			0.01 ng of DNA/rxn	100%	
*T. spiralis*	Larva	Muscle	Mice	Repetitive DNA	X06625	0.724 fg of DNA/rxn	Cross reaction with positive controls, including *Tri. nativa*, *Tri. pseudospiralis* and *Tri. nelsoni*, no cross reaction with heterologous species, no template-free amplification	[95]
						0.002 larvae/rxn		
						0.01 larvae/g of muscle tissue		
				mt-lsrDNA	GU339148.1	0.1 pg/rxn	100%	[96]
*T. canis*	Egg	–	–	ITS-2	AJ002440,	0.1 pg of DNA/rxn	100%	[81]
	Egg	Sand	–			3 eggs/10 g of sand		
	Adult	Stool	Dog			0.1 pg of DNA/rxn	100%	[80]
						3 eggs/30 g of stools		
*T. catti*	Egg	Sand	–	ITS-2	AJ002441	0.1 pg/rxn	100%	[81]
*B. xylophilus*	–	–	–	ITS	AB500146- AB500156 (accessed in DDBJ)	10 copies of DNA/rxn	100%	[97]
	–	Wood	–			2.5 × 10^(−5) of a nematode/rxn	–	
*H. contortus*	Egg	Stool	Sheep	ITS-1	–	5 pg of DNA/rxn	100%	[98]
	Adult	–	Goat	ITS-2	X78803.1	1 pg of DNA/rxn	100%	[99]
Cestodes								
*T. solium*	Proglottid and cysticercus	Cyst fluid	Mouse	Cox1	AB086256	–	100%	[100]
	Proglottid and cysticercus	Cyst fluid	Mouse	Clp	AB441815	1 copy of DNA/rxn	100%	
*T. saginata*	Proglottid and cysticercus	Cyst fluid	Mouse	Cox1	AY684274	–	100%	[100]
	Egg	Stool	Human			5 EPG	100%	
	Proglottid and cysticercus	Cyst fluid	Mouse	Clp	AB441816	1 copy of DNA/rxn	100%	
	Egg	Stool	Human			more than 10 EPG	97.4%(confirmed by multiplex PCR with Cox1 genes)	
*T. asiatica*	Proglottid and cysticercus	Cyst fluid	Mouse	Cox1	AF445798	–	100%	[100]
	Egg	Stool	Human			5 EPG	100%	
*E. granulosus*	Protoscolex	Liver	Sheep	Repeat region sequence	DQ157697	100 fg DNA/200 μl	100%	[105]
	Egg	Stool	Dog			1 pg/200 mg feces	–	
						5 EPG		
	Egg	Stool	Dog	Nad5	AF297617	1 pg of DNA/rxn	100%	[106]
	Egg	Stool	Dog			positive from 22 days p.i. with 10 000 protoscoleces	–	
	Egg and larva	Stool	Dog			10 pg of DNA/rxn	100%	[104]
*E. granulosus* sensu stricto	Protoscolex	–	–	Nad1	AF297617	1/10 or 1/50 of one proscolex	*E. granulosus G1, E. granulosus G3* positive;	[108]
							100%	
*E. equinus*	Protoscolex	–	–	Nad1	AF346403	1/10 or 1/50 of one proscolex	100%	[108]
*E. Canadensis*	Protoscolex	–	–	Nad1	AB208063	1/10 or 1/50 of one proscolex	*E. Canadensis G6, E. Canadensis G7, E. Canadensis G8, E. Canadensis G10* positive;	[108]
							100%	
*E. felidi*	Egg	–	–	Nad1	EF558357	1/10 and 1/50 egg	100%	[105]
*E. ortleppi*	Protoscolex	–	–	Nad1	AB235846	1/10 or 1/50 of one proscolex	100%	[105]
	Protoscolex and associated germinal layer	Hydatid cyst	Camel and human	Nad1	JN637177	10 pg of DNA/rxn	100%	[109]
*E. multilocularis*	Protoscolex	Multilocular cystic masses	Mouse	Nad5	AB031351	1 pg of DNA/rxn	100%	[107]
	Egg	Stool	Dog			positive from 12 days p.i. with 10 000 protoscoleces	100%	
	Larva	–	Human	Cox1	AB46141	1 pg of DNA/rxn	100%	[104]
	Egg	Stool	Dog			5 egg of DNA extraction	100%	
*E. shiquicus*	Adult	–	Fox	Cox1	JF90613	10 pg of DNA/rxn	100%	[104]
*T. hydatigena*	Adult	–	Fox	Cox1	JN83129	10 pg of DNA/rxn	100%	[104]
	Egg	Stool	Dog			1 egg of DNA extraction	100%	
*T. multiceps*	Larva	–	Sheep	Nad1	KC79480	1 pg of DNA/rxn	100%	[104]
	Egg	Stool	Dog			2 egg of DNA extraction	100%	
*T. crassiceps*	Larvae	–	Gerbil	Cox1	EU54454	10 pg of DNA/rxn	100%	[104]
*T. pisiofmi*	Adult	–	Dog	Cox1	JX67796	10 pg of DNA/rxn	100%	[104]

*ITS-1* Internal transcribed spacer 1, *ITS-2* Internal transcribed spacer 2, *IGS* Intergenic spacer, *Cox1* Cytochrome c oxidase subunit 1 gene, *Clp* cathepsin L-like cysteine peptidase, *Nad 1* The mitochondrial NADH dehydrogenase subunit 1 (Nad1) gene, *Nad 5* The mitochondrial NADH dehydrogenase subunit 5 (Nad5) gene, *OvMS6 Opisthorchis viverrini* microsatellite 6, *SjR2 Schistosoma japonicum* retrotransposon 2, *p.i* Post-infection, *EPG* Egg per gram of feces-: unavailable, *Mt* The mitochondrial Nad5 gene, *WbLDR* W. bancrofti Long DNA repeat, *OvGST Onchocerca volvulus* glutathione S-transferase, *RF4* Repeat family 4, *NTC* Non-template Control, *Mt-lsrDNA* The mitochondrial-large subunit ribosomal DNA

### Detection of trematodes by LAMP

Foodborne trematode infections remain a serious global health burden, resulting in 2 million disability-adjusted life years lost annually [110, 111].

Clonorchiasis and opisthorchiasis, being mainly prevalent in Asia and Europe, are characterised by significant pathological hepatobiliary changes caused by *C. sinensis*, *O. viverrini* and *O. felineus* [110, 112]. Both *C. sinensis* and *O. viverrini*, classified as class one carcinogens of human cholangiocarcinoma by the International Agency for Research on Cancer, are cancerogenic after years of infestation in bile ducts of the host [112, 113]. As developed as biotechnology tools are, microscopic egg counting in stool samples continues to be the routine method of diagnosis, which is simple but lacks sensitivity in early and light infections [112, 114, 115]. How to accurately differentiate between liver flukes and intestinal flukes in areas where they coexist remains an unsolved problem [116]. In endemic areas where residents become infected by consuming raw fish with metacercariae, the epidemiological investigation of *C. sinensis* infection in freshwater fish is an important part of clonorchiasis supervision. The current epidemiological method in fish partly depends on the labour-intensive microscopic inspection of fish muscle, which may lead to missed detection of low worm burden or cross-border contamination [117, 118]. Hence, LAMP, as an innovative technique that is sensitive and convenient, will help to solve these problems. The LAMP assay has been devised to detect DNA of *C. sinensis* and *O. viverrini* in freshwater snails [12], the second intermediate fish hosts [14, 60, 61] and patient faeces [26, 61, 62].

In the detection of *C. sinensis* infection in fish, the respective detection limit of LAMP and PCR were 10^− 8^ ng/μL and 10^− 6^ ng/μL, respectively, demonstrating that LAMP was 100-fold more sensitive than PCR [60]. When the true positive and negative results of LAMP were in 100% agreement with the conventional microscopic examination, this approach shows the potential to replace the conventional method in the investigation of fluke invasion in the fish industry [14, 60, 61]. In addition, LAMP is sensitive enough to examine up to 0.0002 cercariae in a snail, and it is promising to be a prominent figure in epidemiological surveillance for snail control intervention [12]. In human faecal samples, LAMP-based technology was established to detect *C. sinensis* with infection intensity as low as 1 egg per 100 mg. Further evaluation of the LAMP-based diagnosis test showed a sensitivity of 97.1% and specificity of 100% as confirmed by the Kato-Katz (KK) method as well as real-time PCR (RT-PCR) [26]. However, it also perceived five additional positive samples of 13 microscopically negative samples in *O. viverrini* determination [61]. Future studies are expected to assess the valid detection limit of this method in comparison with the KK method and RT-PCR as well as its feasibility as a routine standard method [26]. Similar LAMP assays were also developed in *O. viverrini*, with the variation of sensitivity and specificity relating to the repetition of different target genes when detecting copro-DNA [14, 61, 62]. For example, LAMP is highly sensitive when targeting internal transcribed spacer 1 (ITS1) of *O. viverrini*, but specificity cannot be guaranteed for ITS1 cross-amplifying genes from *O. felineus*, *F. gigantica* and *Haplorchoihoides* spp. [61, 62]. When amplifying the mitochondrial gene nad1 of *O. viverrini* in 100% specificity, the sensitivity for LAMP was between 1 petagram (pg) and 100 femtograms (fg), whereas it was 10 pg for PCR [62].

Amphimeriasis, caused by *Amphimerus* spp., has been recently reported as an emerging zoonotic fish-borne trematodisasis affecting indigenous inhabitants and domestic animals in the tropical Pacific side of Ecuador [119]. To date, a novel LAMP assay (namely LAMPhimerus) is devised for the first time to detect internal transcribed spacer 2 (ITS2) of *Amphimerus* spp. DNA in patient faecal samples, with detection limit (1 pg) identical to conventional PCR [63]. LAMPhimerus was more sensitive than traditional parasitological techniques, including direct microscopy detection, formalin-ether concentration, simple sedimentation technique, Kato-Katz technique, fecal egg count [63]. Of 44 human stool samples, the LAMPhimerus method achieved 76.67% sensitivity; 80.77% specificity; 82.14% positive predict value (PPV) and 75.00% negative predict value (NPV) [63]. As the current scarce genomic information of *Amphimerus* spp. is scarce, further enhancement of the assay could be based on the exploitation of different DNA target [63]. The procedure, in combination with the air-dried faecal specimens on common filter paper as source of DNA, is superior in feasible collection, long-term preservation and transportation, and potentially applicable as an effective diagnostic or epidemiological tool in amphimeriasis-endemic regions [64]. Furthermore, the system ‘air-dried stool sample on filter paper’-LAMP assay would be practical in large-scale molecular investigation of the other helminthiasis [64].

Given the infection of the genus *Fasciola*, fascioliasis mainly affects ruminants and only occasionally humans, raising public health and economic concerns due to a reduction in output [120–122]. Triclabendazole-resistant *F. hepatica,* an emerging problem, calls for reliable assessment of efficacy or resistance after deworming therapy [122]. Serological ELISA is applied in the detection of cattle and sheep, but it is unreliable for species distinction and the effectiveness of drug therapy [123]. Coproantigen ELISA is appropriate for monitoring adult infection, whereas it is insufficient correlation with larval stage invasion until 6 weeks post treatment [124]. LAMP targeting ribosomal intergenic spacer seems to be an optional detection method that overcomes the difficulty in taxonomical classification of *F. hepatica* and *F. gigantica*. It can amplify genes from adults, eggs and juvenile stages with a sensitivity 10 000-fold higher than PCR, while running an hour faster in the laboratory [65]. Other LAMP-based assays amplifying sequences of the second internal transcribed spacer (ITS2) show their inability to distinguish between the two *Fasciola* species, *F. hepatica* and *F. gigantica* [66, 67]. Under field conditions, the LAMP assay can identify infected sheep in the first week post-infection and 30 days post-therapy, while ELISA cannot detect infections until 6 weeks and is insufficient to discriminate current and past infections, indicating the practical and applicable determination of drug efficacy or resistance [66]. In contrast, M.I. Arifin et al. reported poor performance of LAMP and PCR in comparison with other conventional methods for the diagnosis of *F. hepatica* in naturally infected sheep and cattle in the field. Of the 64 animals examined, LAMP and PCR had low sensitivities of 17.9 and 10.7%, respectively, and high specificities of 97.2 and 100%, respectively, with faecal egg count (FEC) and coproantigen ELISA as composite reference standards. The failure of LAMP and PCR may be due to factors including insufficiency of DNA sample, possibly in relation to the choice of DNA extraction method, amount of faeces substantially used, and uneven egg distribution in faeces of different host species [67]. If promoted in the future, such a test is still suitable for early diagnosis, thus reducing veterinary costs and the loss of livestock due to fascioliasis [65–67]. To the best of our knowledge, LAMP has not yet been used for the detection of human fascioliasis.

Paragonimiasis, also known as lung fluke disease, is a pulmonary inflammation caused by *Paragonimus* species [125, 126], of which *P. westermani* is the most epidemiologically relevant in Asia and sporadically in American and African countries [127]. The conventional immunological diagnosis method is sensitive in human paragonimiasis but unsustainable in epidemiological surveys when intermediate hosts are detected [128]. A LAMP assay has successfully amplified the gene sequence of *P. westermani* eggs in sputum and pleura fluid from patients, as well as metacercariae in freshwater crabs and crayfish. With a detection limit of 1 × 10^− 8^ ng/μL, LAMP is close to 100 times more sensitive than PCR. The LAMP method also yields positive and negative results coinciding with those from parasitology tests, acting as an excellent candidate for field surveys and clinical diagnoses of paragonimiasis [15].

Schistosomiasis ranks on the list of neglected tropical diseases (NTD) for its impacts on an estimated number of over 200 million individuals in more than 70 countries [126, 129, 130]. Of the five *Schistosoma* spp. that usually cause human schistosomiasis, *S. japonicum* is prevalent in Asia, while *S. mansoni* and *S. haematobium* are mainly concurrent in Africa and the Middle East [130]. Currently, infection and reinfection continue to be global challenges, particularly in poverty-stricken and insanitary communities [131, 132] and in other regions due to transmission by tourists and immigrants who come into contact with infested water [130, 132]. Meanwhile, low-density infection remains after deworming programmes, which still demands an affordable diagnostic approach for pre-patent infection and massive epidemiological surveillance despite current parasitological, immunological and molecular diagnostic methods [131–134]. The KK method is the current mainstay of schistosomiasis diagnosis, and its drawback of day-to-day variation is inevitable in massive surveillance [9, 130, 131, 134]. In addition, it is of great importance to overcome the limitation of serological methods and their incapacity to discriminate between past and present infections due to the persistent existence of circular antibodies in the patient even after an effective cure [135].

As the control of intermediate host snails considerably contributes to the monitoring of schistosomiasis [126], LAMP assays were established to detect *S. japonicum* in *Oncomelania hupensis* [27, 68], *S. mansoni* in *Biomphalaria* spp. [13, 71, 72, 75] and *S. haematobium* in other snails [71, 72]. LAMP assays are sensitive and specific in pooled samples, with a detection limit of up to one positive in 100 negative *O. hupensis* (expecting for a larger sample) [68] as well as one snail infected with *S. mansoni* in 1000 normal snails [13]. In addition, a snail invaded by a single miracidium can be detected only 1 day after exposure [68, 72, 132]. Therefore, LAMP was used to construct the risk map of schistosomiasis based on infected *O. hupensis* in a field survey and readily adapted to predict the prevalence tendency [27]. What’s more, there is another work of LAMP (named SmMIT-LAMP) assessing not only infected snails but also human stool in low-transmission area of *S. mansoni* in Brazil, where the incidence was corresponded to what has been reported, ascertaining the foci of schistosomiasis transmission and helping build risk maps of schistosomiasis [77]. Furthermore, LAMP was developed to detect *S. japonicum* in rabbit models [16, 69, 70] and *S. mansoni* in murine models [71, 73, 74]. This approach detected positive results as early as 1 week [16, 69], and even 3 days, after low-intensity infection in rabbit models [70], tested negative as late as 12 weeks post treatment, which is consistent with PCR in early diagnosis, and tested negative 2 weeks later than PCR [70], thereby possessing potential in early diagnosis, treatment and assessment of the efficacy after chemotherapy [16, 69, 70]. LAMP is also readily adopted in the clinical determination of *S. japonicum* in human serum samples [16, 70], *S. mansoni* in stool samples [77], as well as *S. mansoni* and *S. haematobium* in urine samples [51, 76]. In human sera with light to mediate infection, LAMP achieves the sensitivity, specificity, PPV and NPV of 95.5, 100, 100 and 89.4%, respectively, whereas those for *S. mansoni* and *S. haematobium* in urine sample are 90–100% [76]. Additionally, the sensitivity (92.86%), specificity (80.11%), and NPV (99.33%) of SmMIT-LAMP in human stool samples are overall acceptable, but the PPV is 26.00%, which can be explained by the higher sensibility of LAMP over the reference standard (KK), especially in patient with low infection levels [77]. In addition, without any need for costly laboratory instrumentation and highly skilled personnel, the refinement of DNA extraction (i.e., LAMPellet, NaOH and heat lysis [51]), the harness of a portable plasma separator [136] and the utility of a user-friendly chip [74] fulfil the requirements of the POC test and are estimated to have a competitive per-person cost, with less than $7.25 for the circulating cathodic antigen test and no more than $7.00 for a single KK test [74]. Accordingly, further evaluation is required for POC use in endemic areas [51, 74, 76].

### Detection of nematodes by LAMP

Nemathelminthiasis, caused by nematodes, is a globally rampant parasitic disease. The pathogenic nematode infecting human includes STH, *S. stercoralis, Toxocara* spp., filariae, and other nematodes with distinctive life cycles, namely, *A. cantonensis* and *Trichinella*. Nematodes in veterinary and agricultural fields are also included.

STH, including *A. lumbricoides*, hookworms, and whipworms, mainly occur in tropical and subtropical regions [137]. The KK method is currently the most common method in STH diagnosis and is recommended by the WHO to conduct STH surveys [17, 78, 79, 138]. However, for the false negative results brought about by the reduction of egg production after chemotherapy or the hatching of eggs due to the delay of examination [139, 140], it is actually a suboptimal choice in a mass drug administration (MDA) programme where post-chemotherapy evaluation is needed. In contrast, the LAMP assay is superior to the parasitological and unspecific serological approaches in that it tests positive when there is merely a single ovum [17], without cross reactivity or non-template positive [17, 78, 79]. In terms of the quantity of DNA, the SmartAmp2 assay amplifies the STH β-tubulin gene provided that there is one pg of DNA [79], and hookworm detection targeting the ITS-2 gene can even succeed with 0.4 fg of DNA [78]. None of the false positives is observed in these LAMPs, which is important, as multiple helminthiases may coexist in individuals in endemic areas [17]. In simulated clinical samples, the LAMP assays exhibit great agreement with the KK method in which the kappa coefficient is calculated to be 0.72 for *A. lumbricoides* determination targeting ITS-1 [79] and 0.9 for hookworm measuring targeting ITS-2 [17, 78]. In the SmartAmp2 assay, the pre-addition of HNB dye achieves even better accuracy by providing a closed system to avoid contamination in post-reaction manipulation using SYBR Green [79]. Bovine serum albumin was added, and it performs well in crudely prepared stool samples despite the presence of inhibitors, which is undoubtedly a competitive advantage for a POC tool, though it still needs further comparison [79]. However, the vulnerability of HNB to pH changes may be a challenge for its stability but can be resolved by standardizing reaction conditions [79].

*S. stercoralis,* acting as one of the opportunistic nematodes transmitted by soil*,* is the causative agent of human strongyloidiasis. It usually contributes to asymptomatic infection but is a deadly uncontrolled hyperinfection syndrome in immunocompromised patients [141–145], with a mortality rate of up to 87% [146, 147]. There is no single gold standard for its detection, as the microscopic examination of larvae in stool samples is insufficiently sensitive even when supplemented with enrichment techniques. Serological tests are sensitive but lack specificity [148–151]. PCR-based techniques, though sufficiently specific, are not diagnostically superior to parasitological techniques because of their unsatisfactory sensitivity, which is presumably attributed to the irregular larval output in chronic strongyloidiasis, the uneven distribution in stool specimens, the DNA extraction process, the existence of inhibitors in stool samples, etc. [151]. Generally, the definitive diagnosis of strongyloidiasis is made by parasitological examinations based on clinical symptoms, serological evidence, etc. [52, 82]. Compared with morphological examination, nucleic acid tests are advantageous in that they can detect specimens where parasites had been killed [52]. In 2014, the LAMP assay for *S. stercoralis* was first reported to be capable of amplifying less than ten 0 DNA copies of larvae per reaction, or 10^− 2^ dilution of one spiked larva in stool samples, comparable to the results of PCR [52]. Unfortunately, the foregoing factors that may influence PCR-based techniques, e.g., the DNA extraction process, also may impact it [52]. Aiming at surmounting the shortcomings of common stool samples, urine samples from rodent models were used in a novel LAMP assay named Strong-LAMP [82]. The creative introduction of urine samples may possess predominant advantages in collection, storage and processing over stool samples. Furthermore, when employing urine samples of the rodent model, Strong-LAMP shows positive results from 5 days after infection of 40 third-stage (L3) infective larvae (1 day earlier than employing stool samples) to 3 days after infection of 400 or 4000 L3 infective larvae (2 days earlier than employing stool samples). Nevertheless, since requests for urine samples in *S. stercoralis* detection are rare, its clinical value in latent infection of humans needs further study [82].

The larvae of *T. canis* and *T. cati* are responsible for human toxocariasis. Children specifically tend to acquire these kinds of telluric zoonosis and saprozoonosis by environmental exposure to *Toxocara* spp. [152], which makes it one of the most common cosmopolitan helminthiases [153]. The prevention of its transmission depends on the condition of the environmental contamination levels and the accurate determination of its sources [81]. However, *Toxocara* identification by traditional microscopy of stools from pets or environmental samples remains a methodological concern due to its insensitivity in low-burden cases and its difficulty in distinguishing *T. canis* from *T. cati* eggs [80, 81]. PCR assays have been designed to discern *Toxocara* spp. in stools [154] or environmental samples [155] and to distinguish between *T. canis* and *T. cati* in soil samples [156]. The species-specific LAMP assay targeting ITS-2 was validated by two groups and found to be ten-fold more sensitive than PCR without cross reactivity in the laboratory between *Toxocara* spp. and is applied in domesticated dogs and sand samples [80, 81]. In the context of environmental specimens, LAMP manifests a detection limit of 3 eggs/10 g of sand and less than 3 eggs/30 g of stools, compared with the 6 eggs/10 g of sand and more than 2 eggs/30 g of stools detection limit of PCR [80, 81]. In a field survey of soil contamination, LAMP yield a positive rate of 42.7% versus 7.7% of PCR [157]. In another field study, even LAMP fails to identify very low contamination, which is a pitfall that may be attributed to the crude processing of DNA extraction in LAMP compared with that of PCR [81], the LAMP assay successfully decreased the standard examination time by 50% compared to that of PCR [81].

As one of the most debilitating infectious diseases in the world, lymphatic filariasis, which is caused by brugian filariae and *W. bancrofti*, is also regarded as a serious public health concern for 856 million people in 52 countries around the world [158]. The WHO MDA programme effectively reduces morbidity, raising new concerns about diagnosis and surveillance in the control areas and determination of the treatment endpoint in the post-MDA stage [8, 83, 87, 88, 159]. So far, the diagnosis largely counts on the microfilaraemia test, which employs night blood samples [86, 88] and is recommended by the WHO to conduct a transmission assessment survey (TAS) where *Brugia* spp. is endemic. It is used as the minimum in TAS but suffers from the reduction of sensitivity in response to the prevalence decrease in the post-MDA era. Simultaneously, more accurate methods, such as antibody tests and PCR, are restricted by their inherent shortcomings. The antigenaemia tests recommended to map *W. bancrofti* endemicity, namely, immunochromatography card test and filariasis test strip [160, 161], are unavailable for brugian filariae and may cross react with *Loa loa* [160, 162, 163]. Alternatively, as a competitive candidate in the present study, LAMP assays manifest cheerful outcomes in both laboratory and clinical tests [87, 88]. For instance, the *W. bancrofti* LAMP test, with a determination limit of 0.1 pg per reaction equivalent to that of PCR, costs over $1.38 less than the latter [87]. It is estimated that there is approximately 200 pg and 100 pg of DNA inside a single microfilaria of *W. bancrofti* or *Brugia* spp., respectively [164]; that is to say, the detection limit of the LAMP assay exceeds the theoretical detection limit of microfilariae per ml via microscopic inspection [165]. Furthermore, compared with the serological tests that are inadequately specific, almost all the LAMP assays for lymphatic filaria diagnosis are species-specific, except one detecting brugian filariae for both *B. timori* and *B. malayi* [86–88].

A similar methodological handicap is used to eliminate *O. volvulus*, another major public health concern mostly rampant in sub-Saharan Africa [83, 166]. Following the impediment to onchocerciasis transmission, the challenge emerges in that the conventional diagnostic method of skin snip microscopy and the primary diagnostic antibody test, the Ov-16 rapid diagnostic test, is losing its sensitivity in low-prevalence settings [167, 168]. Alternatively, nucleic acid-based assays can be employed in both diagnosis and xenomonitoring with extreme sensitivity and specificity. O-150 PCR, therefore, is recommended by the WHO to undertake vector surveillance but is limited in resource-limited areas [84, 169]. Using the economical LAMP assay as a diagnostic option manifests sensitivity just slightly lower than the utmost sensitive qPCR when targeting cox1 but is ten times higher than conventional PCR in O-150 assay at the same time [84, 85]. In terms of specificity, the cox1 assay is reported to cross react with *O. chengi*, a sympatric cattle parasite transmitted by black flies, or rather, the cox1 assay can be used only in clinical diagnosis using skin biopsy samples unless significant progress is made to improve specificity [85]. However, whether the other set of primers designed for O-150 can amplify the heterologous sequence from *O. chengi* remains to be determined [84], as the PCR targeting O-150 has been proven to cross react with *O. chengi* unless a specific DNA probe is added [170]. In addition, an elaborate comparison is designed between the HNB and neutral red dyes, and the latter improves the sensitivity 10-fold, which sheds light on a new approach for parasite LAMP amelioration, maximizing its usefulness in a world with a changing global landscape of infection [84].

In contrast to other parasites, in the post-MDA surveillance of filariae, the exploitation of samples from mosquito vectors is considered timelier, more operationally feasible and more ethically accepted than detection using specimens from humans [8, 159, 168, 169, 171]. As entomological inspection via field dissension is expensive, time consuming and unable to distinguish *O. volvulus* from *O. chengi,* O-150 PCR using vector samples is currently widely accepted to determine the interruption of filariae [8, 87, 159, 167–169]. LAMP can also act as an excellent surrogate for PCR in this case. As shown in *O. volvulus* detection targeting OvGST1a, without crossreactivity with *O. chengi* or other filariae, LAMP tests positive with merely 0.01 ng of DNA spiked in 200 insects, which is more sensitive than PCR, which tests positive in 0.01 ng/50 insects [83]. Based on the conventional LAMP assays, an improved non-instrumented nucleic acid-LAMP was developed, devised as a single portable electricity free device with comparable or even higher sensitivity than a normal assay, demonstrating that it is more suited for field surveys [86]. Whereas the existing LAMP assays for vector monitoring are designed to utilize the DNA extracted from infective-stage larvae (L3), there are great hurdles in xenomonitoring, where the DNA test cannot identify DNA from L3 larvae from immature stage parasites (L1 or L2) in vectors, which actually distinguishes xenomonitoring from entomological monitoring of transmission [159]. As the discrimination between infectious and immature parasites will clarify whether the positive result is due to adult filariae not responding to drug treatment or recent infection indicating active transmission, it is taking on increasing significance in the assessment after large-scale drug treatment [8, 171]. For *O. volvulus,* in which the infectious stage parasites are located in the head capsule isolated from the immature stage larvae in the abdomen and thoracic muscle, the obstacle can be overcome by the separation of the head and body and therefore provide accurate evaluation of transmission [159, 172]. On the other hand, although there are specific L3-stage RT-PCR tests that are capable of indirectly determining the infection potential and transmission dynamics of lymphatic filariae via RNA [173, 174], dissection remains more common for the detection of infectious stage lymphatic filariae [159]. However, it can be expected that the development of RT-LAMP in parasitology may favour this technique to replace RT-PCR and conventional dissection to precisely predict the transmission potential even in low-resource areas.

*Loa loa* is a long-neglected filariae that is reported to cause deadly serious adverse events after ivermectin treatment [86, 89–91, 175, 176] at a low threshold of microfilaria (mf) burden [175], where determination of the mf burden before MDA programme is especially important. Unfortunately, the routine diagnosis and quantification in remote areas rely on microscopic inspection of midday blood samples, which requires expertise and processing of a considerable number of samples and is unqualified to serve as a POC or a large-scale screening tool. Among the existing LAMPs, one amplifies the LL3M9 gene and exhibits the lowest detection limit of 0.5 ag/reaction, far lower than the formerly reported 0.1 pg/reaction for *W. bancrofti* [87, 90]. Considering the practical significance of *Loa loa* mf burden quantitation in MDA practice, *Loa loa* LAMP targeting LLMF72 was assessed for its potential for semi-quantitation. As a result, a correlation was observed between the time to LAMP reaction positivity (minutes) and the mf concentration in the blood, allowing the naked-eye determination of whether the mf burden is above or below the specific threshold. For example, the run time to positivity is 15 min at the threshold of > 30 000 mf/mL, 20 min at threshold of > 5000 mf/mL, and 25 min at the threshold of >v100 mf/mL, which is promising for application in the *Loa loa* microfilaraemia assessment before ivermectin treatment and thus facilitating the elimination of filariasis [89]. Since the LL3M9 comprises multiple copies of a simple nematode-conserved repeat, and LLMF72 is a single copy gene, which may exert an impact on the sensitivity and specificity, a new bioinformatic pipeline is designed to mine a new species-specific sequence that is more suitable for MDA practice. Consequently, RF4 is a new biomarker with specificity; however, it lacks sensitivity compared with the LL3M9 or LLMF72 assays. Nevertheless, the bioinformatic pipeline remains a creative and robust method to further explore the potential of LAMP [91].

Dirofilariasis caused by *D. repens*, another species of mosquito-borne filariae [177], is regarded as an emerging zoonotic disease calling for more accurate diagnosis. The traditional diagnostic method relies on microscopic examination of blood from the hosts [178]. Serological screenings [179] and PCR tests have been designed [180, 181]. The LAMP assay targeting the COI gene was devised as 2 versions for further evaluation. With respect to sensitivity, the detection limits of reverse transcriptase LAMP (RT-LAMP) and propidium iodide LAMP (PI-LAMP) are 0.15 fg and 10 fg, respectively, versus the detection limit of 15 fg for conventional PCR. With a lower limit, the LAMP assays yield amplicons within approximately 40 min, while conventional PCR takes 2 h. Generally, both versions of LAMP prevail over conventional PCR in both sensitivity and efficiency, while all of them are species-specific in the current study. Considering practical value, while RT-LAMP employs a RT-PCR instrument, PI-LAMP, by introducing propidium iodide, permits visualization of the amplification as UV fluorescence, meriting more widespread application in field surveys and clinical diagnoses [92]. Because of its combination of sensitivity, specificity, rapidity and convenience, it may be a promising ancillary tool in dirofilariasis surveillance and prevention, such as large-scale travelling animal quarantine inspection or culicid mosquito screening.

*A. cantonensis* infects people on the Pacific islands and Southeast Asia. It is the major cause of an eosinophilic meningitis in humans in endemic areas [182]. The lack of standardization of a diagnostic procedure and the current situation of being overlooked in accounts for the use of a presumptive diagnosis, which is primarily based on the combination of patient history and clinical criteria, e.g., morphological examination of adult worms or larvae in cerebrospinal fluid, of which the positive rate is between 2%~ 12% [183], are unable to meet the expectation of either clinical diagnosis or large-scale surveillance [184, 185]. In an effort to help establish a surveillance system, two LAMP assays were developed to detect the L3 larvae in molluscan hosts. One amplifying the ITS-1 gene manifests a detection limit of 1 fg/reaction [94]. The other test targeting the 18S rRNA gene is inferior, with a detection limit of 10 pg/reaction [93], while both have higher sensitivity than PCR, which can detect DNA > 100 pg/reaction [93, 94]. In a similar field survey, the ITS-1 LAMP assay demonstrates detection rates of 6.7 and 4.4% higher than the standard digestion method and PCR, respectively [94]. In summary, all of the above information exhibits considerable potential and superiority in replacing existing approaches in large-scale field surveys and clinical diagnoses [93, 94].

Trichinellosis is a significant zoonotic disease caused by the ingestion of raw or insufficiently cooked meat containing *Trichinella* spp., for which the inadequacy of veterinary control is one factor to blame. There had been no detailed and systematic reports of the sensitivity and conditions of the assays for *Trichinella* determination by 2012, when 2 LAMP assays were designed [95, 96], amplifying mitochondrial large ribosomal subunit DNA (mt-lsrDNA) and a 1.6 kb repetitive sequence from the larvae, respectively. Both assays manifest sensitivity 10-fold stronger than conventional PCR [95, 96], but the one targeting mt-lsrDNA turns out to be 10-fold less sensitive than RT-PCR [96]. Further exploration could be made to improve the sensitivity of LAMP to make it an optimal methodology for trichinellosis detection in practice, e.g., meat quarantine or field survey.

In addition to the human medical nematoda mentioned above, the application of LAMP has spread to the veterinary [98, 99] and agricultural fields [97], which makes it a promising detection tool shared by all fields of bioscience.

### Detection of cestodes by LAMP

*Taenia* species (*T. solium, T. saginata* and *T. asiatica*), the causative pathogens of taeniasis, can be sympatrically endemic in Asia, such as in China and Thailand [186]. *T. solium,* normally transmitted between pigs and humans, results in neurocysticercosis with a range of manifestations, especially epilepsy and seizures [7]. Conventional proglottid examination, as a common diagnostic method for taeniasis, fails to morphologically differentiate the eggs of *Taenia* species*.* Multiplex PCR and nested PCR open the door for characteristic discrimination [187, 188] but are unrealistically applied in field surveys for high expense and time considerations. Therefore, a LAMP assay with the cytochrome c oxidase subunit 1 (cox1) primer set was developed for the differentiation of *Taenia* spp*.* at the species level in the laboratory and in the field, managing to detect eggs in traditional faecal samples in epidemiological surveys with high specificity and even higher sensitivity than PCR [100–103]. Ranging from five to ten eggs per gram (EPG) of faeces, the detection limit of LAMP is comparable to that of five EPG and 40 EPG of multiplex PCR and nested PCR, respectively [100, 187, 188]. The specificity is approximately 100%, with only two in 76 (2.6%) *T. saginata* recognized as *T. asiatica* in faecal samples [100]. Out of 51 proglottids expelled from 35 carriers, consistent results were obtained by LAMP under field conditions and in the laboratory, except for one sample [102]. Thus, the tedious procedure of simultaneously identifying *Taenia* species is expected to be simplified to reduce the possibility of cross contamination and to save time, while the handy copro-DNA extraction method is expected to take the place of centrifugation. Remarkably, the modification of mLAMP combined with dot-ELISA has succeeded in specific amplification in a single tube, demonstrating an easier and more practical POC diagnostic method for real-time human *Taenia* species confirmation [44].

Widely distributed in pastoral areas worldwide but often neglected, echinococcosis, especially cystic echinococcosis and alveolar echinococcosis, attracts enormous attention by posing as a threat to both humans and animals and results in economic loss [189–193]. An on-site approach is expected to replace the ethically-challenged post-mortem inspection as the gold standard in susceptible *Echinococcus*-infected definitive canid hosts [189, 193]. In addition, a more practical and available tool is sought to solve the problem of copro-ELISA lacking sensitivity in latent infection monitoring [194] and to sustain reliability of copro-PCR while reducing the expense [195, 196] in epidemiological surveillance in endemic areas at the same time. LAMP was exploited to detect *E. granulosus s. s.* (G1-G3) copro-DNA in dogs [104–106] and then cysts in camels and humans [109]. It stands out for its high sensitivity in detecting infection in copro-samples from definitive hosts 22 days after exposure, which is equivalent to 3 days, 4 days and 47 days earlier than ELISA, conventional PCR and light microscopy, respectively [106]. A similar advance in *E. multilocularis* determination depicts LAMP as a substantial alternative for field surveillance of AE in areas of endemicity [107]. LAMP was also applied in other cestodes of veterinary relevance, including *E. equinus* (G4), *E. canadensis* (G6-G10), *E. felidi* (lion strain), *E. ortleppi* (G5) [108], *E. shiquicus, T. hydatigena, T. multiceps, T. pisiformis* and *T. crassiceps* [104]. Furthermore, it was sensitive enough to distinguish different *Echinococcus* species, achieving sensitivity down to 2% of a single protoscolex or egg per reaction [104, 108], but failed to discriminate at the genotype level [108]. There are not yet inadequate data to relate intrastrain genetic variants to different life cycles, pathogenicity or any other practical relative features [191, 192, 194, 197, 198]. Subsequently, LAMP has great potential to become a new tool for future perspectives on molecular epidemiology in echinococcosis surveillance at this stage. Additionally, the real-time LAMP assay gave 100% concordance with the results obtained by nested RT-PCR when testing parasite DNA extracted from hydatid cysts from domestic animals and humans, which highlights a brilliant future in clinical diagnosis of CE [108, 109]. Recently, LAMP was first reported to determine *Taenia* species in an epidemiological survey in Mongolia [199]. Above all, the rapid, sensitive and accurate LAMP is sufficient to facilitate a large-scale epidemiological survey.

### Application of LAMP in field research

As discussed above, the LAMP assay is a robust and versatile tool that is capable of meeting the WHO’s requirements for ideal POC tools of ASSURED and possesses the potential to become an appealing option for field research, which was substantiated by a series of laboratory and diagnostic tests.

From the perspective of field application, major achievements were made for LAMP assays for malaria and tuberculosis [200, 201]; in both cases, scientists worked extensively with the WHO for the implementation of the tests in field, and their standardized reagent kits have been used in developing countries as patient side tools [202]. For protozoa, bacteria and fungi, several commercial reagent kits have been put on the market and have performed excellently [203, 204]. With respect to helminths, considerable significance is attached to filariae. LAMP assays for the detection filariae have already come to MDA management practice in Guinea, Nigeria and Southeast Asia [205–207]. In a recent epidemiological survey in Mongolia, LAMP also played a significant part [199].

## Conclusions

To sum up, though presently in its infancy, the LAMP assay is a groundbreaking DNA amplification technique with prominent advantages. Its ASSURED characteristics and its versatility in adapting to various circumstances make it an ideal POC tool and friendly to field surveys. The chief shortcoming of LAMP is the false-positive result caused by primer-primer reaction and contamination. The former needs further evaluation, and the latter can be solved by the amelioration of the reaction system, detection approaches, etc. Another handicap in LAMP development is the difficulty in primer design. However, its merits outweigh its weakness, and LAMP has blossomed in the detection of microorganisms and protozoa detection and has already entered into the market and epidemiological surveys. Overall, the methodology will be improved in the future, and the active role of LAMP in clinical and epidemiological practice is foreseeable.

## Additional file

Additional file 1: Multilingual abstract in the five official working languages of the United Nations. (PDF 590 kb)

## Abbreviations

ASSURED: Affordable, Sensitive, Specific, User-friendly, Rapid and equipment-delivered
ELISA: Enzyme-linked immunosorbent assay
EPG: Egg (or eggs) per gram
FIP: Forward inner primer
HNB: Hydroxy naphthol blue
KK: Kato-Katz
LAMP: Loop-mediated isothermal amplification
MDA: Mass drug administration
mf: Microfilariae
mLAMP: Multiplex LAMP
NPV: Negative predict value
NTD: Neglected tropical diseases
PCR: Polymerase chain reaction
PEI: Poly-ethylenimine
POC: Point-of-care
PPV: Positive predict value
RT-PCR: Real-time PCR
STH: Soil-transmitted helminth
TAS: Transmission assessment survey
