# Supplementary material for: Detection of helminths by loop-mediated isothermal amplification assay: a review of updated technology and future outlook
Source: Infect Dis Poverty. 2019 Mar 25;8:20. doi: 10.1186/s40249-019-0530-z (PMC6432754; doi:10.1186/s40249-019-0530-z)
Supplement: Supplementary file 1 — Multilingual abstract in the five official working languages of the United Nations. (PDF 590 kb) [file 40249_2019_530_MOESM1_ESM.pdf]

Translation of the abstract into the five official working languages of the United Nations

إكتشاف الديدان الطفيلية بإستخدام إختبار **LAMP**. مراجعة من التكنولوجيا الحديثة والعلوم المستقبلية.

Okanurak Kamolnetr, Yanin Limpanont, Zhiyue Lv ,Miaohan Deng, Lanyi Zhong

الملخص
خلفية عامة : الديدان الطفيلية تستوطن أكثر من نصف المدن حول العالم ، والتي تثير مخاوف خطيرة على الصحة العامة . التشخيص الدقيق حول الديدان الطفيلية هو أمر بالغ الأهمية. الطرق التقليدية في علم الطفيليات للكشف عن الديدان الطفيلية هو عباره عن :١- فحوص مصلية ٢- PCRوهذه الطرق التقليدية تعتمد فقط على الفحوصات لإكتشاف الديدان الطفيلية ، وهذا يستغرق الوقت الكبير جداً . وأحيانا تكون نتائج تلك الفحوصات ليست دقيقة كافية . فحص LAMP هو عبارة عن طريقة مطورة حساسة سريعة وبسيطة لإكتشاف الديدان الطفيلية .الهدف من هذه الدراسة هو لمناقشة مدى تأثير LAMP في إكتشاف الديدان الطفيلية ، ومقارنة بين التكنولوجيا الحديثة والعلوم المستقبلية مع طرق التشخيص الآخرى .
الفكرة الرئيسية : هي تلخيص مدى تأثير إختبارLAMP في إكتشاف الديدان الطفيلية و مراقبة داء الديدان الطفيلية . المبدأ الأساسي ل LAMP هو تقديم المساعدة لفهم أفضل للخصائص ، ويتم تقييم كل اختبار ذكرت استنادا على أساس الإكتشاف الحساس والدقيق والمقيد مقارنة مع الفحوصات الآخرى .
علاوة على ذلك ؛ نخن نناقش بعض قيود الفحوصات لتوضيح بعض الطرق المحتملة للتحسين .
الخلاصة : نحن نلخص ونناقش المزايا والعيوب . ونتطلع إلى مستقبل واعد من فحص LAMP في إكتشاف الديدان الطفيلية ، وهو المتوقع أن يقدم المساعدة في تطوير المعرفة الحالية ووجهات النظر المستقبلية عن فحص LAMP. إختبار LAMP هو حساس للغاية في تشخيص داء الديدان الطفيلية وأيضاً يستعد في إكتشاف بعض الأمراض الطفيلية الآخرى، والذي يمكن أن يساهم في القضاء على الأمراض من المناطق الموبوءة .

Translated from English version into Arabic by Ali K M Daiesh

用环介导等温扩增技术检测蠕虫：一篇关于最新技术及其前景的综述

邓淼菡，钟兰仪，Okanurak Kamolnetr, Yanin Limpanont，吕志跃

摘要
引言：蠕虫流行于世界上超过一半的国家，导致严重的公共卫生问题，而对蠕虫感染的准确诊断是蠕虫控制策略的关键。传统病原学方法、血清学方法以及基于多聚酶链式扩增技术的各种检测方法是目前蠕虫感染的常见检测手段，但昂贵、费时，且有时检测结果不准确。环介导等温扩增技术是近期出现的一种敏感、简单、快速的方法，已被越来越多的研究者用于蠕虫检测。本研究旨在讨论环介导等温扩增技术在蠕虫检测中应用的现状，并通过与其他诊断方法的比较，对这种新型技术及其应用发展前景进行综合评估。
主要内容：本综述概述了应用于蠕虫检测以及蠕虫病监测的环介导等温扩增技术。介绍了环介导等温扩增技术的基本原理，以帮助读者更好地理解其特性；通过对比其他主要检测手段，对现有应用环介导等温扩增原理的蠕虫检测方法进行敏感性、特异性及自身局限性的评价。此外，本文还对环介导等温扩增技术应用蠕虫检测的局限性及其潜在的改进方法进行了讨论。
结论：本文总结和讨论了环介导等温扩增技术在蠕虫检测中的优势、缺点及其良好应用前景，了解其在蠕虫病及其它寄生虫病的高敏感性、高特异性诊断与监测中的最新进展和发展前景，将有助于这些寄生虫病的控制与消除。

Translated from English version into Chinese by Zhi-yue Lv, Miao-Han Deng, Lan-Yi Zhon

Détection des helminthes par un essai d'amplification isotherme à médiation par boucles: une révision de nouvelles technologies et perspectives d'avenir

Miaohan Deng, Lanyi Zhong, Okanurak Kamolnetr, Yanin Limpanont, Zhiyue Lv

Résumé
Contexte : Les helminthes sont endémiques dans plus de la moitié des pays du monde, et suscitent de graves préoccupations en santé publique. Le diagnostic précis de la présence des helminthes est déterminant en matière de stratégies de surveillance. Les méthodes parasitologiques classiques, les tests sérologiques et les analyses fondées sur la réaction en chaîne de la polymérase (PCR) représentent les principaux modes de diagnostic de la présence des helminthes, mais ils s'avèrent chronophages et/ou onéreux et donnent parfois des résultats imprécis. L'essai d'amplification isotherme à médiation par boucles (LAMP) est une méthode sensible, simple et rapide qui a été développée afin de détecter les helminthes. Cette étude a pour objectif de discuter de l'état actuel d'application de

l'essai LAMP dans le cadre de la détection des helminthes et d'effectuer une évaluation approfondie de cette nouvelle technologie et de ses perspectives d'avenir, en le comparant à plusieurs autres méthodes diagnostiques.
Corps principal : La présente révision résume l'essai LAMP appliqué à la détection des helminthes et à la surveillance des helminthiases. Le principe de base de l'essai LAMP est présenté afin de mieux comprendre ses caractéristiques et chaque essai reporté est évalué principalement sur la base de sa sensibilité, de sa spécificité et de ses limites par rapport aux autres tests de diagnostic courants. Par ailleurs, nous discutons des limites des essais afin de clarifier certaines possibilités d'amélioration.
Conclusions : Nous récapitulons et discutons ici des avantages, des inconvénients et des perspectives prometteuses de l'essai LAMP dans le cadre de la détection des helminthes, qui devrait contribuer à mettre à jour les connaissances actuelles et les futures perspectives de l'essai LAMP dans le diagnostic hautement sensible et spécifique ainsi que dans la surveillance des helminthiases et d'autres maladies parasitaires, et pourrait aider à l'élimination des maladies dans les zones endémiques.

Translated from English version into French by Eric Ragu, proofread by Chrystelle Colette Ngateu, through

Обнаружение гельминтов с помощью петлевой изотермической амплификации: обзор новейшей технологии и прогноз на будущее

Мьяохан Дэн, Ланьи Чжун, Оканурак Камолнетр, Янин Лимпанонт, Чжийю Лв

Аннотация

Справочная информация: Заражение гельминтами (глистами) является эндемичным для более чем половины стран мира и является серьезной проблемой с точки зрения здоровья населения. Точная диагностика заражения гельминтами необходима для выработки стратегии сдерживания эндемий. Традиционные паразитологические методы, серологические исследования и ПЦР-анализы являются основными средствами диагностики гельминтов, однако указанные методы являются дорогостоящими и/или требуют много времени, а иногда дают неточные результаты. Для обнаружения гельминтов был разработан анализ с помощью петлевой изотермической амплификации (LAMP), представляющий собой точный, простой и быстрый метод диагностики. Цель данного исследования - рассмотреть нынешний статус применения LAMP для обнаружения гельминтов и дать всестороннюю оценку этой новейшей технологии и ее перспектив путем сравнения с несколькими другими методами диагностики.
Основная часть: В данном обзоре кратко изложена информация о применении петлевой изотермической амплификации (LAMP) для обнаружения гельминтов и эпидемиологического надзора за гельминтозом. Прежде всего дается объяснение основного принципа петлевой изотермической амплификации (LAMP) для того, чтобы легче было понять ее характеристики, при этом каждый описываемый анализ оценивается в основном с точки зрения его точности, специфичности и ограничений по сравнению с другими общепринятыми методами диагностики. Кроме того, в работе рассматриваются ограничения и недостатки описываемых анализов, для того, чтобы прояснить некоторые возможные пути усовершенствования.
Выводы: Здесь мы подводим итог и обсуждаем преимущества, недостатки и многообещающее будущее петлевой изотермической амплификации в обнаружении гельминтов, что поможет дополнить имеющиеся знания и улучшить перспективы использования LAMP для чрезвычайно чувствительной и специфической диагностики и надзора за гельминтозом и другими паразитарными заболеваниями, а также может способствовать их искоренению в регионах, для которых они являются эндемичными.

Translated from English version into Russian by Ludmila Minkov, proofread by Michael Orlov, through

Detección de helmintos mediante la técnica de amplificación isotérmica mediada por bucle: una revisión de la tecnología actual y las perspectivas de futuro

Miaohan Deng, Lanyi Zhong, Okanurak Kamolnetr, Yanin Limpanont, Zhiyue Lv

Resumen

Contexto: los helmintos son endémicos en más de la mitad de los países del mundo, lo que plantea graves problemas de salud pública. Un diagnóstico preciso de los helmintos es crucial para las estrategias de control. Los métodos parasitológicos tradicionales, las pruebas serológicas y los ensayos basados en la PCR son los principales medios de diagnóstico de los helmintos, pero llevan mucho tiempo o son caros, y a veces proporcionan resultados inexactos. La técnica de amplificación isotérmica mediada por bucle (LAMP, por sus siglas en inglés) es un método sensible, simple y rápido que fue desarrollado para la detección de helmintos. El objetivo de este estudio es discutir el estado actual de la aplicación de la LAMP en la detección de helmintos y realizar una evaluación exhaustiva sobre esta tecnología actual y sus perspectivas futuras comparándola con otros métodos de diagnóstico.

Texto principal: esta revisión resume la técnica LAMP aplicada para la detección de helmintos y la vigilancia de la helmintiasis. El principio básico de la LAMP se introduce para ayudar a comprender mejor sus características y cada uno de los ensayos presentados se evalúa principalmente basándose en su sensibilidad de detección, especificidad y limitaciones, en comparación con otras pruebas de diagnóstico comunes. Además, discutimos las limitaciones de los ensayos para aclarar algunas posibles formas de mejorarlos.

Conclusiones: aquí resumimos y discutimos las ventajas, desventajas y el futuro prometedor de la LAMP en la detección de helmintos, que se espera que ayude a actualizar el conocimiento actual y las perspectivas futuras de la LAMP en el diagnóstico y vigilancia altamente sensibles y específicos de la helmintiasis y otras enfermedades parasitarias, y que puede contribuir a la eliminación de las enfermedades en las zonas endémicas.

Translated from English version into Spanish by Lia Sarra Felip, proofread by Mayra León, through

TRANSLATORS
WITHOUT BORDERS
